# Reframing Post‐Hepatectomy Liver Failure as a Determinant of Post‐Recurrence Survival in HCC

**DOI:** 10.1002/jhbp.70087

**Published:** 2026-02-11

**Authors:** Hiroshi Horie, Satoshi Ogiso, Tomoaki Yoh, Takahiro Nishio, Ken Fukumitsu, Yoichiro Uchida, Takamichi Ishii, Etsuro Hatano

**Affiliations:** ^1^ Department of Surgery, Graduate School of Medicine Kyoto University Kyoto Japan

**Keywords:** hepatocellular carcinoma, liver failure, liver function, recurrence, salvage therapy

## Abstract

**Background:**

Post‐hepatectomy liver failure (PHLF) is traditionally considered a transient perioperative complication with limited long‐term consequences, yet its long‐term oncologic implications in hepatocellular carcinoma (HCC) remain unclear.

**Methods:**

Patients undergoing primary hepatectomy for HCC between 2007 and 2018 were analyzed. To ensure reliable assessment of recurrence and post‐recurrence survival, follow‐up was continued through 2023. Associations between PHLF and overall survival (OS), time to recurrence (TTR), and survival after recurrence (SAR) were assessed. Propensity score matching (PSM) analysis controlled for baseline differences.

**Results:**

PHLF ≥ grade B occurred in 59 patients (14.6%). It was significantly associated with worse OS (45.6 vs. 109.7 months, *p* < 0.001) and SAR (21.5 vs. 55.9 months, *p* < 0.001), but not TTR. After PSM, these associations persisted. At recurrence, patients with PHLF had worse ALBI scores (−1.92 vs. –2.57, *p* < 0.001) and lower rates of re‐resection for recurrent disease (6.9% vs. 29.3%, *p* = 0.026), even after PSM.

**Conclusions:**

PHLF ≥ grade B compromised hepatic reserve at recurrence and limiting curative salvage therapy, leading to impaired SAR and OS. Prevention of PHLF should be prioritized not only to reduce perioperative risk but also to optimize long‐term oncologic outcomes in HCC.

## Graphical abstract

Horie and colleagues showed that post‐hepatectomy liver failure ≥ grade B significantly compromised survival after recurrence in hepatocellular carcinoma by impairing hepatic reserve and limiting curative salvage options. This adverse impact persisted after propensity score matching, reframing post‐hepatectomy liver failure as a key modifiable determinant of long‐term oncologic outcomes.

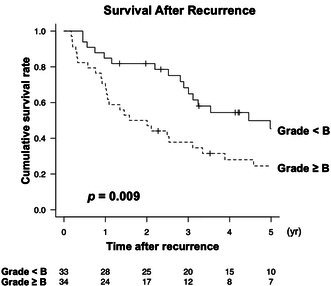

**Abbreviations**
ALBI: Albumin‐Bilirubin
HCC: hepatocellular carcinoma
INR: international normalized ratio
ISGLS: International Study Group of Liver Surgery
MTA: molecular targeted agents
OS: overall survival
PHLF: post‐hepatectomy liver failure
PSM: propensity score matching
RFS: recurrence free survival
SAR: survival after recurrence
TACE: transcatheter arterial chemoembolization
TTR: time to recurrence

## 1 Introduction

Hepatocellular carcinoma (HCC) is one of the most prevalent malignancies and ranks as the third‐leading cause of cancer‐related deaths worldwide [1]. Hepatectomy remains a cornerstone treatment for HCC, but the majority of patients eventually experience recurrence in more than 10% of patients annually, with cumulative recurrence rates reaching 70%–80% at 5 years [2]. In this context, survival after recurrence (SAR) has gained attention as a critical determinant of long‐term prognosis [3, 4, 5, 6].

The ability to undergo curative salvage therapies such as re‐resection or ablation at the time of recurrence largely depends on preserved liver function. Post‐hepatectomy liver failure (PHLF) is a well‐recognized complication that may impair hepatic reserve. According to the standardized definition proposed by the International Study Group of Liver Surgery (ISGLS), PHLF is defined as an increase in serum bilirubin and prolongation of INR on or after postoperative day 5 compared with preoperative values, with reported incidence ranging from 1% to 32% [7, 8]. While Grade C PHLF requires invasive interventions such as hemodialysis, intubation, or reoperation and carries high mortality typically precluding long‐term survival, Grade B PHLF is often considered a mild and reversible condition, treated with non‐invasive supportive measures such as daily administration of diuretics or albumin and mild respiratory support. Consequently, PHLF—especially Grade B—has traditionally been regarded as a short‐term issue without lasting impact on oncologic outcomes. However, whether such “mild” PHLF compromises liver function at the time of recurrence and limits eligibility for curative salvage treatments remains unknown.

To address this gap, we aimed to investigate the association between PHLF and long‐term outcomes, with a particular focus on SAR, using propensity score–matched analysis in a single‐center cohort with long‐term follow‐up.

## 2 Patients and Methods

### 2.1 Patients

We retrospectively reviewed the prospectively maintained institutional database of the department of surgery at Kyoto university. The study protocol was approved by the ethics committee of the Kyoto university hospital. The patients who underwent primary hepatectomy for HCC with curative intent between January 2007 and December 2018 were included in the study to ensure a sufficiently long follow‐up duration for the evaluation of recurrence treatments and survival after recurrence. Follow‐up data were updated through December 2023 to capture late recurrences and long‐term outcomes. Clinicopathological data at hepatectomy and at initial recurrence, as well as treatment and survival data, were retrieved. The occurrence of PHLF was evaluated based on the definition by ISGLS: Grade A: significant increase in the international normalized ratio (INR) and bilirubin levels on day 5 after surgery but requiring no change in the clinical management of the patient; Grade B: abnormally elevated INRs and bilirubin levels resulting in a deviation from regular clinical management but manageable without invasive treatment; and Grade C: similar to grade B but requiring invasive treatment [8]. Liver function was assessed using the albumin‐bilirubin (ALBI) score and grading system [9]. The modified ALBI grading system was also used for a more detailed evaluation of liver function [10]. The pathologic fibrosis stage of the resected liver was evaluated according to the METAVIR score [11]. Macrovascular invasion was defined as the presence of a tumor thrombus in the second‐order branches or more central segments of the portal vein, and/or in the main trunk of the right, middle, or left hepatic vein, or the inferior vena cava [12]. Patients with missing data at hepatectomy or recurrence and those who underwent salvage liver transplantation were excluded. Liver transplantation fundamentally alters hepatic function and prognosis, making it inappropriate to evaluate the prognostic impact of liver function at recurrence within the framework of this study. OS, time to recurrence (TTR), and SAR were evaluated as major endpoints to investigate the impact of PHLF on oncologic prognosis.

### 2.2 Hepatectomy

Our surgical strategy for HCC was reported previously [13]. The indications for hepatectomy included a CP grade of A or B, and indocyanine green clearance of remnant liver (ICG‐Krem) < 0.03 was defined as a contraindication [14]. The extent of resection was determined based on the parenchyma‐sparing concept, and anatomic resection was not mandatory if the surgical margins could be secured. Major hepatectomy was defined as resection of three or more Couinaud liver segments, in accordance with the Brisbane 2000 terminology and widely accepted clinical definitions [15, 16]. The Pringle maneuver was routinely performed to reduce blood loss during parenchymal transection.

### 2.3 Follow‐Up Protocol

All patients were followed up every 3–4 months after hepatectomy. Physical examination, liver function tests, tumor markers, and imaging studies including ultrasonography, computed tomography, and magnetic resonance imaging were performed as appropriate.

### 2.4 Treatments for Recurrence

Treatment strategies for recurrent HCC were determined in a multidisciplinary tumor board, considering factors such as recurrence pattern, tumor size and number, anatomical location, liver function, time to recurrence, availability of alternative treatments, and patient performance status.

In the case of intrahepatic lesions, percutaneous local ablation therapies such as radiofrequency ablation were primarily chosen for small tumors (≤ 2 cm) or a limited number (3 or fewer) of tumors. Transarterial therapies like transcatheter arterial chemoembolization (TACE) were favored for multiple (3 or more) recurrences. Liver re‐resection was considered for tumors larger than 2 cm or those resistant to nonsurgical treatments.

Regarding extrahepatic lesions, re‐resection was the only curative treatment and was indicated in very select patients exhibiting technically resectable recurrences. Systemic chemotherapy with molecular targeted agents (MTAs) was indicated when patients developed extrahepatic recurrence and when prior treatment with ablation therapy or TACE was ineffective for intrahepatic recurrence. Patients with bone or brain metastases who were not candidates for surgery were treated with radiotherapy.

### 2.5 Statistical Analysis

Baseline characteristics were presented using standard descriptive statistics: medians (25th and 75th percentiles) for continuous variables and percentages for categorical variables. The Mann–Whitney U test was performed for continuous variables, and the *χ* ^2^ or Fisher's exact test was used for categorical variables as appropriate. Kaplan–Meier curves were generated according to PHLF grade (No PHLF, A, B or C and PHLF ≥ grade B or PHLF < grade B). OS and SAR were compared using the Kaplan–Meier method and the log‐rank test. Cumulative incidence of post‐hepatectomy recurrence was compared using Gray's test. Any deaths were set as competing risk of recurrence in the analyses. Propensity score matching (PSM) was performed to adjust for both preoperative and intraoperative confounders to examine the causal impact of PHLF on long‐term survival. A propensity score was calculated using a logistic regression model. All preoperative variables (age, sex, viral hepatitis, ALBI score, AFP level, tumor diameter, multiple tumors, poor differentiation, cirrhosis, macrovascular invasion, major hepatectomy), with or without intraoperative variables (operative time, blood loss, and margin status), were included in the propensity score model. After the propensity score was generated, patients in PHLF ≥ B or < B groups underwent 1:2 nearest‐available matching of the logit of the propensity score with a caliper width of 0.10 of the standard deviation of the score. Statistical significance was set at *p* < 0.05. All statistical analyses were performed using R version 4.1.0 (R Foundation for Statistical Computing, Vienna, Austria).

## 3 Results

### 3.1 Patients' Characteristics at Hepatectomy and at Recurrence in the Entire Cohort

**FIGURE 1:**
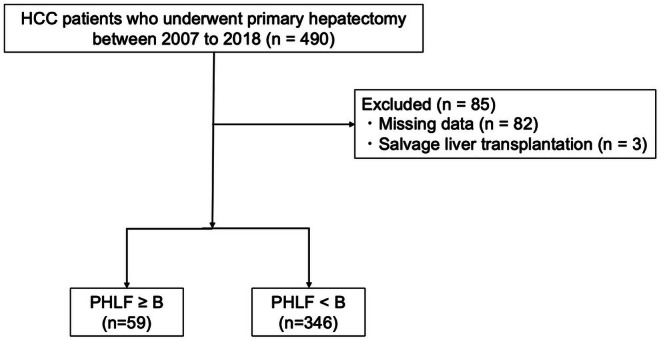
The flow chart of this study.

During the study period, 490 patients underwent hepatectomy for HCC, of whom 405 met the inclusion criteria and were analyzed. Among them, 59 patients experienced PHLF ≥ grade B, while 346 had PHLF < grade B (Figure 1). Clinicopathological characteristics at the time of primary hepatectomy and at the time of recurrence are summarized in Table 1. Post‐hepatectomy recurrence was observed in 234 patients (57.8%). At the time of initial hepatectomy, cirrhosis (F4) (*p* = 0.017) was significantly more common in patients who developed PHLF ≥ grade B. Patients with PHLF ≥ grade B had a significantly higher proportion of poorer mALBI grades (*p* = 0.003). Median operation time was significantly longer (*p* = 0.004), and operative blood loss was significantly greater (*p* = 0.003) in patients who developed PHLF ≥ grade B. At the time of recurrence, patients with PHLF ≥ grade B had significantly higher ALBI scores and poorer mALBI grades (*p* < 0.001 for both) and underwent re‐resection less frequently than those with PHLF < grade B (*p* = 0.005).

**TABLE 1:** Clinicopathological characteristics at hepatectomy and at recurrence according to PHLF grade (≥ B vs. < B).

Primary hepatectomy	PHLF < grade B	PHLF ≥ grade B	*p*	SD
	*n* = 346	*n* = 59		
Preoperative findings				
Age > 70 years., *n* (%)	160 (46.2)	22 (37.3)	0.257	0.182
Sex, male, *n* (%)	272 (78.6)	49 (83.1)	0.492	0.113
Viral hepatitis, *n* (%)	200 (57.8)	40 (67.8)	0.155	0.208
ALBI score, median (IQR)	−2.62 (−2.90 to −2.53)	−2.53 (−2.75 to −2.26)	0.062	0.273
mALBI grade I, *n* (%)	189 (54.6)	20 (33.9)		
grade IIa, *n* (%)	104 (30.1)	16 (27.1)		
grade IIb, *n* (%)	51 (14.7)	23 (39.0)		
grade III, *n* (%)	2 (0.6)	0 (0.0)	0.003*, †	
Serum AFP levels > 200 ng/mL, *n* (%)	90 (26.0)	14 (23.7)	0.872	0.053
Tumor diameter > 5 cm, *n* (%)	105 (30.3)	20 (33.9)	0.648	0.076
Multiple tumors, *n* (%)	85 (24.6)	21 (35.6)	0.080	0.242
Poor differentiation, *n* (%)	84 (24.3)	15 (25.4)	0.870	0.027
Cirrhosis (F4), *n* (%)	112 (33.9)	29 (50.9)	0.017*	0.348
Macrovascular invasion, *n* (%)	40 (11.7)	8 (13.8)	0.663	0.062
Major hepatectomy, *n* (%)	123 (35.5)	27 (45.8)	0.146	0.209
Perioperative findings				
Operation time (min), median (IQR)	361.0 (275.3–457.8)	427.0 (310.5–515.5)	0.004*	
Blood loss > 1000 mL, *n* (%)	87 (25.2)	27 (45.8)	0.003*	
R1 margin, *n* (%)	25 (7.3)	2 (3.5)	0.400	
**Recurrence**	***n* = 198**	***n* = 36**		
Findings at recurrence				
Age > 70 years., *n* (%)	86 (43.4)	14 (38.9)	0.715	
Serum AFP levels > 200 ng/mL, *n* (%)	11.2 (22.0)	7 (19.4)	0.174	
ALBI score, median (IQR)	−2.57 (−2.82 to −2.35)	−1.96 (−2.34 to −1.75)	< 0.001*	
mALBI grade I, *n* (%)	94 (47.5)	6 (16.7)		
grade IIa, *n* (%)	63 (31.8)	4 (11.1)		
grade IIb, *n* (%)	36 (18.2)	26 (72.2)		
grade III, *n* (%)	5 (2.5)	0 (0.0)	< 0.001*, †	
Beyond Milan criteria, *n* (%)	59 (29.8)	17 (47.2)	0.052	
Treatment for recurrence				
Ablation therapy, *n* (%)	89 (44.9)	11 (30.6)	0.143	
Re‐resection, *n* (%)	54 (27.3)	2 (5.6)	0.005*	
Re‐resection of IHR, *n* (%)	40 (20.2)	1 (2.8)	0.008*	
Re‐resection of EHR, *n* (%)	16 (8.1)	2 (5.6)	1.000	
Transarterial therapy, *n* (%)	111 (56.1)	26 (72.2)	0.097	
MTA, *n* (%)	69 (34.8)	12 (33.3)	1.000	

Abbreviations: AFP, alpha‐fetoprotein; ALBI score, albumin‐bilirubin score; EHR, extrahepatic recurrence; IHR, intrahepatic recurrence; IQR, interquartile range; mALBI grade, modified albumin‐bilirubin grade; MTA, molecular targeted agent; PHLF, post‐hepatectomy liver failure; SD, Standardized Difference.

* Statistically significant difference.

^†^ Fisher's exact test; Bonferroni‐corrected pairwise comparisons showed significant differences between Grade IIb and Grade I or IIa.

### 3.2 Overall Survival, Time to Recurrence and Survival After Recurrence

**FIGURE 2:**
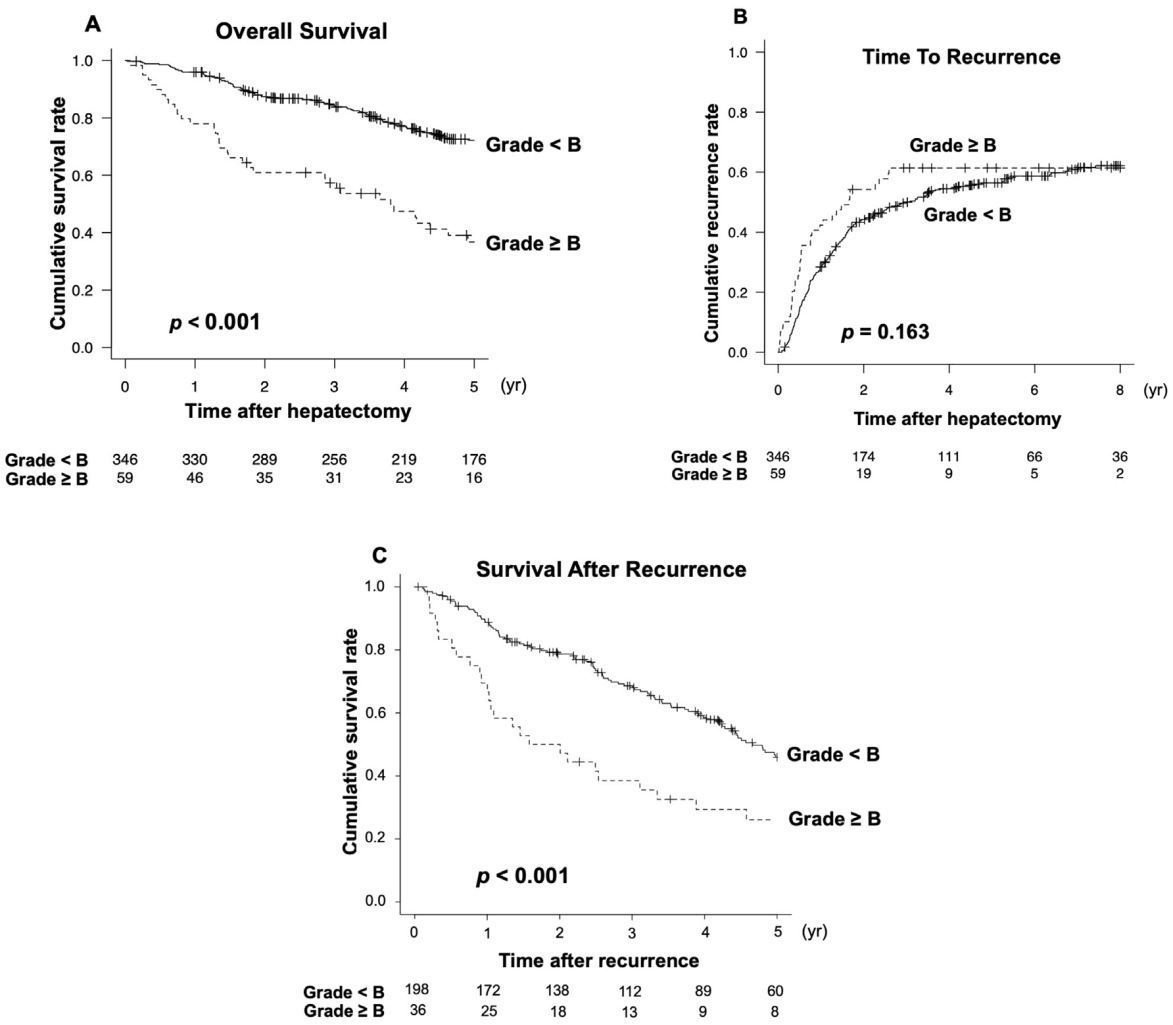
Kaplan–Meier curves for overall survival (A), time to recurrence (B), and survival after recurrence (C) in patients with PHLF grade ≥ B versus < B, as shown by Kaplan–Meier analysis.

Analyses of OS, TTR, and SAR between the two groups are presented in Figure 2. Both OS and SAR were significantly shorter in the PHLF ≥ grade B group compared to the PHLF < grade B group. Specifically, the median OS was 45.6 months in the PHLF ≥ B group versus 109.7 months in the < B group, with corresponding 1‐, 3‐, and 5‐year OS rates of 78.0%, 57.3% and 36.7% versus 95.9%, 84.1% and 72.2%, respectively (Hazard ratio, 2.68; 95% CI, 1.89–3.81; *p* < 0.001). The median SAR was 21.5 months versus 55.9 months, and the 1‐, 3‐, and 5‐year SAR rates were 69.4%, 38.5%, and 26.1% versus 88.7%, 68.6%, and 45.9%, respectively (Hazard ratio, 2.21; 95% CI, 1.48–3.30; *p* < 0.001). TTR did not differ significantly between the two groups (17.7 versus 41.9 months, *p* = 0.163). Thus, the observed differences in overall survival were primarily driven by post‐recurrence outcomes, not initial recurrence timing. Additional analyses of OS and SAR stratified by each PHLF grade are shown in Figure S1.

### 3.3 Patients' Characteristics and Long‐Term Survival in the Propensity Score–Matched Cohort

**FIGURE 3:**
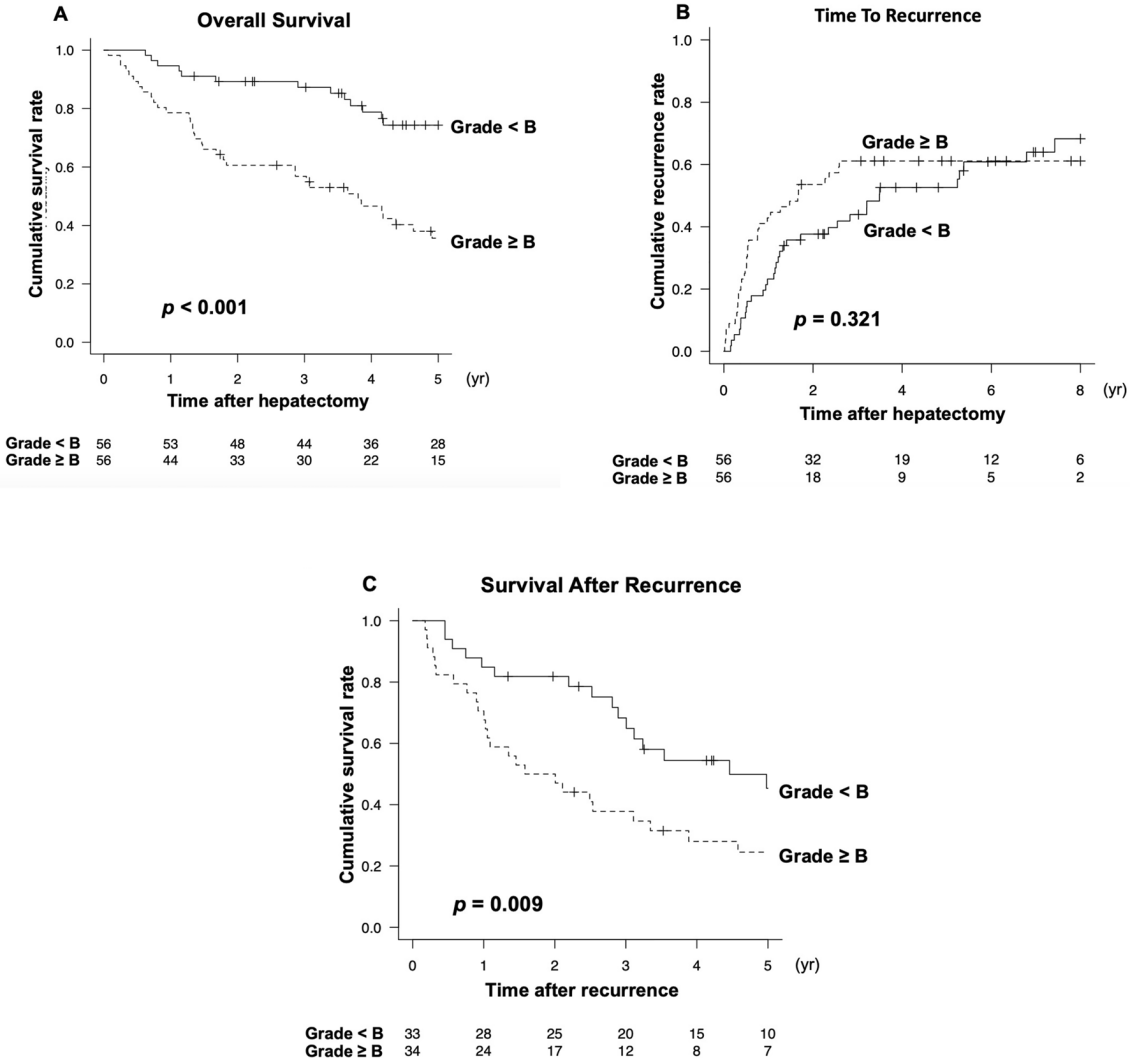
Kaplan–Meier curves for overall survival (A), time to recurrence (B), and survival after recurrence (C) in patients with PHLF grade ≥ B versus < B, after propensity score matching based on preoperative variables.

Using PSM with preoperative variables, 50 patients with PHLF > B were matched with 100 patients with PHLF ≤ B. The clinicopathological characteristics after matching are summarized in Table 2. The absolute values of the standardized differences for all preoperative variables were within 0.2, indicating an adequate quality of matching. After PSM, Median operation time and the proportion of intraoperative blood loss > 1000 mL was significantly higher in the PHLF ≥ grade B group (*p* = 0.004 and 0.003, respectively). At recurrence, liver function was significantly worse in the PHLF ≥ grade B group, as indicated by higher ALBI scores (−1.92 vs. −2.57, *p* < 0.001) and more advanced mALBI grades (*p* < 0.001). Patients in the PHLF ≥ grade B group underwent re‐resection for recurrent lesion less frequently (6.9% and 29.3%, *p* = 0.026), particularly for intrahepatic recurrence (3.4% vs. 25.9%, *p* = 0.013). In contrast, the proportion of patients who underwent transarterial therapies was significantly higher in the PHLF ≥ B group (79.3% vs. 51.7%, *p* = 0.019). Survival analyses in the matched cohort are shown in Figure 3. Both OS and SAR were significantly shorter in the PHLF ≥ grade B group. The median OS was 45.6 months versus 128.2 months (Hazard ratio, 2.86; 95% CI, 1.78–4.58; *p* < 0.001), and the medians SAR was 24.1 months versus 59.8 months (Hazard ratio, 2.45; 95% CI, 1.42–4.23; *p* = 0.001). In contrast, there was no significant difference in TTR between the groups (20.2 versus 41.9 months, *p* = 0.526).

**TABLE 2:** Characteristics after propensity score matching on preoperative variables (PHLF ≥ B vs. < B).

Primary hepatectomy	PHLF < grade B	PHLF ≥ grade B	*p*	SD
	*n* = 100	*n* = 50		
Preoperative findings				
Age > 70 years., *n* (%)	45 (45.0)	22 (44.0)	1.000	0.020
Sex, male, *n* (%)	84 (84.0)	42 (84.0)	1.000	0.000
Viral hepatitis, *n* (%)	65 (65.0)	33 (66.0)	1.000	0.024
ALBI score, median (IQR)	−2.53 (−2.83 to −2.22)	−2.60 (−2.73 to −2.29)	0.989	0.020
mALBI grade I, *n* (%)	45 (45.0)	26 (52.0)		
grade IIa, *n* (%)	24 (24.0)	12 (24.0)		
grade IIb, *n* (%)	30 (30.0)	12 (24.0)		
grade III, *n* (%)	1 (1.0)	0 (0.0)	0.784	
Serum AFP levels > 200 ng/mL, *n* (%)	28 (28.0)	10 (20.0)	0.325	0.188
Tumor diameter > 5 cm, *n* (%)	37 (37.0)	17 (34.0)	1.000	0.063
Multiple tumors, *n* (%)	22 (39.3)	20 (35.7)	0.857	0.043
Poor differentiation, *n* (%)	30 (30.0)	11 (22.0)	0.337	0.183
Cirrhosis (F4), *n* (%)	46 (46.0)	24 (48.0)	0.863	0.040
Macrovascular invasion, *n* (%)	15 (15.0)	7 (14.0)	1.000	0.028
Major hepatectomy, *n* (%)	49 (49.0)	22 (44.0)	0.606	0.100
Perioperative findings				
Operation time (min), median (IQR)	373.5 (278.3–482.3)	429.0 (345.5–549.5)	0.03*	
Blood loss > 1000 mL, *n* (%)	27 (27.0)	25 (50.0)	0.007*	
R1 margin, *n* (%)	8 (8.0)	2 (4.2)	0.500	
**Recurrence**	***n* = 58**	***n* = 29**	***p* value**	
Findings at recurrence				
Age > 70 years., *n* (%)	29 (50.0)	11 (37.9)	0.363	
Serum AFP levels > 200 ng/mL, *n* (%)	8 (13.8)	3 (10.3)	0.745	
ALBI score, median (IQR)	−2.57 (−2.85 to −2.36)	−1.92 (−2.17 to −1.74)	< 0.001*	
mALBI grade I, *n* (%)	28 (48.3)	4 (13.8)		
grade IIa, *n* (%)	22 (37.9)	3 (10.3)		
grade IIb, *n* (%)	7 (12.1)	22 (75.9)		
grade III, *n* (%)	1 (1.7)	0 (0.0)	< 0.001*, †	
Beyond Milan criteria, *n* (%)	18 (31.0)	12 (41.4)	0.350	
Treatment for recurrence				
Ablation therapy, *n* (%)	25 (43.1)	8 (27.6)	0.241	
Re‐resection, *n* (%)	17 (29.3)	2 (6.9)	0.026*	
Re‐resection of IHR, *n* (%)	15 (25.9)	1 (3.4)	0.016*	
Re‐resection of EHR, *n* (%)	4 (6.9)	2 (6.9)	1.000	
Transarterial therapy, *n* (%)	30 (51.7)	23 (79.3)	0.019*	
MTA, *n* (%)	20 (34.5)	8 (27.6)	0.629	

Abbreviations: AFP, alpha‐fetoprotein; ALBI score, albumin‐bilirubin score; EHR, extrahepatic recurrence; IHR, intrahepatic recurrence; IQR, interquartile range; mALBI grade, modified albumin‐bilirubin grade; MTA, molecular targeted agent; PHLF, post‐hepatectomy liver failure; SD, Standardized Difference.

* Statistically significant difference.

^†^ Fisher's exact test; Bonferroni‐corrected pairwise comparisons showed significant differences between Grade IIb and Grade I or IIa.

Using PSM incorporating both preoperative and intraoperative variables, 48 patients with PHLF ≥ B were matched with 96 patients with PHLF < B (Table S1). At recurrence, liver function was still significantly worse in the patients with PHLF ≥ grade B, as indicated by higher ALBI scores (−1.94 vs. −2.67, *p* < 0.001) and more advanced mALBI grades (*p* < 0.001). Patients with PHLF ≥ grade B underwent re‐resection for recurrent lesions less frequently than those with PHLF < grade B (6.5% and 25.4%, *p* = 0.030), especially for intrahepatic recurrence (3.2% and 19.4%, *p* = 0.034). Patients who underwent transarterial therapies were significantly higher in the PHLF ≥ B group (71.0% vs. 47.8%, *p* = 0.049). Survival analyses in the matched cohort are shown in Figure S2. They also had significantly shorter median OS (45.6 months, not applicable in PHLF < B group; Hazard ratio, 2.988; 95% CI, 1.85–4.84; *p* < 0.001) and median SAR (17.4 months vs. 67.9 months; Hazard ratio, 2.72; 95% CI, 1.60–4.60; *p* < 0.001), while their median TTR was not significantly different (15.2 months vs. 29.5 months; *p* = 0.428).

## 4 Discussion

This study demonstrated that PHLF ≥ grade B has a profound negative impact on long‐term outcomes in patients undergoing curative resection for HCC. In particular, we found that PHLF significantly compromised SAR, independent of its influence on TTR. Our findings highlight an underappreciated consequence of PHLF: even in cases classified as grade B—traditionally considered mild and reversible—hepatic reserve may not fully recover in the long term, limiting the feasibility of potentially curative salvage treatments such as re‐resection or RFA. In our cohort, patients who developed PHLF ≥ grade B had significantly lower rates of curative re‐treatment and worse ALBI scores at recurrence, which likely contributed to impaired SAR.

PHLF has traditionally been viewed as a short‐term postoperative complication, with its long‐term implications receiving limited attention. Once patients recover from PHLF, it has been assumed that long‐term survival is not significantly affected. As such, little attention has been paid to the possibility that PHLF may compromise post‐recurrence liver function and ultimately limit eligibility for curative treatments upon recurrence. Our data challenge such conventional perception and suggest that early PHLF may have latent effects that only manifest later, when recurrence occurs and the opportunity for curative salvage is lost.

Notably, recurrence‐free survival (RFS) has shown minimal improvement over time, and no established surgical or adjuvant strategies effectively prevent recurrence [17]. In contrast, SAR has markedly improved due to advances in locoregional and systemic therapies, and it now correlates more closely with OS [4, 5, 18]. These developments highlight the limitations of using RFS or DFS as the sole endpoint for evaluating long‐term outcomes. In contemporary HCC care, where recurrence is common and salvage therapies are increasingly effective, post‐recurrence management plays a decisive role in determining patient prognosis. However, perioperative events like PHLF have been mostly evaluated in terms of OS or DFS, with little attention paid to their subsequent impact on SAR [19, 20, 21]. To address this gap, our study specifically examined the influence of PHLF not only on TTR, but also on liver function and treatment feasibility at the time of recurrence. Although PHLF ≥ grade B had no significant effect on TTR, it was associated with a 2.13‐fold increased risk of post‐recurrence death. Mechanistically, impaired liver function following PHLF compromised the feasibility of curative reinterventions—particularly repeat resection for intrahepatic recurrence—and led to a treatment shift toward non‐curative modalities such as TACE, ultimately resulting in inferior SAR [3, 4]. This shift was likely driven by treatment eligibility rather than by tumor burden alone. These findings are consistent with accumulating evidence that preserved liver function is a critical determinant of prognosis in HCC, even in the era of molecular targeted agents and immunotherapies [22, 23].

In our cohort, PHLF occurred in 22.5% of patients, with 64.8% classified as grades B or C—consistent with previous reports [24]. Preoperative liver function is a well‐established predictor of PHLF [25, 26], and this association was reaffirmed using the ALBI score, a sensitive and reliable indicator of hepatic reserve [10, 27]. PSM analysis showed that even with comparable preoperative liver function, patients with PHLF ≥ grade B exhibited significantly poorer ALBI scores at recurrence, indicating sustained hepatic impairment. In addition to baseline liver function, intraoperative parameters such as prolonged operative time and increased blood loss have also been recognized as key contributors to PHLF [28, 29, 30]. This association was again confirmed in our cohort matched on preoperative variables. While these intraoperative factors represent modifiable targets through surgical strategies, our PSM analysis including intraoperative variables underscores that PHLF itself imposes a lasting adverse impact on liver reserve and survival—independently of perioperative metrics.

Taken together, our findings emphasize that PHLF should no longer be considered solely a perioperative complication. Rather, it is a modifiable determinant of long‐term outcomes that compromises liver function at a critical therapeutic juncture—recurrence—when eligibility for effective salvage therapies is essential. Preventing PHLF thus holds dual significance: reducing short‐term surgical morbidity and preserving the option for effective treatment upon recurrence. This paradigm shift positions PHLF prevention as a key strategy in optimizing both perioperative and long‐term oncologic outcomes.

This study has several limitations that should be acknowledged. First, its retrospective, single‐center design is inherently subject to selection bias and unmeasured confounding. In addition, a substantial number of patients were excluded due to missing data, which may limit generalizability. Although propensity score matching was applied to minimize bias from known and clinically relevant variables, residual confounding cannot be entirely excluded.

Second, treatment selection at recurrence was multifactorial and influenced not only by hepatic functional reserve but also by technical factors such as tumor location, remnant liver anatomy, surgical complexity, and physician judgment, reflecting institution‐specific practice patterns. In this context, patients who underwent salvage liver transplantation after primary hepatectomy were excluded; however, only three such cases were identified, precluding meaningful statistical evaluation. Given that salvage liver transplantation represents a distinct therapeutic strategy with unique eligibility criteria and prognostic implications compared with liver‐preserving treatments, its impact on survival after recurrence could not be assessed.

Third, although we observed a strong association between PHLF ≥ grade B and impaired liver function at recurrence, the retrospective design does not allow definitive conclusions regarding irreversibility or causality. Other factors, including progression of underlying liver disease, aging, and time to recurrence, may also have contributed to hepatic functional decline.

Finally, the long study period raises the possibility that advances in post‐recurrence treatments, including molecular targeted agents and immunotherapies, may have influenced survival after recurrence. Nevertheless, although the surgical cohort spanned up to 2018, the study was designed to assess long‐term post‐recurrence survival, and follow‐up data were collected until 2023. This extended follow‐up period enhances the reliability of SAR assessment, particularly in the era of evolving treatment strategies for recurrent HCC.

In conclusion, PHLF ≥ grade B is not merely a postoperative complication but a critical determinant of recurrence management and long‐term survival in HCC. By impairing liver function at recurrence, PHLF limits curative treatment options and worsens both SAR and OS. These findings underscore the importance of preventing PHLF as a core objective in liver surgery—vital not only for improving perioperative recovery but also for sustaining the path toward long‐term survival.

## Funding

The authors have nothing to report.

## Ethics Statement

Approval of the Research Protocol by a Institutional Reviewer Board: This study was approved by the ethics committee of the Kyoto University Hospital—approval: R1721.

## Consent

All adult participants provided written informed consent to participate in this study.

## Conflicts of Interest

The authors declare no conflicts of interest.

## Supporting information


**Data S1:** Supplementary Information.

## Data Availability

The data that support the findings of this study are available from the corresponding author upon reasonable request.
