# Supplementary material for: Reframing Post‐Hepatectomy Liver Failure as a Determinant of Post‐Recurrence Survival in HCC
Source: J Hepatobiliary Pancreat Sci. 2026 Feb 11;33(5):343–51. doi: 10.1002/jhbp.70087 (PMC13206420; doi:10.1002/jhbp.70087)
Supplement: Supplementary file 1 — Data S1: Supplementary Information. [file JHBP-33-343-s001.docx]

**Supplemental data**

**Title**

Reframing Post-Hepatectomy Liver Failure as a Determinant of Post-Recurrence Survival in HCC

**Authors**

Hiroshi Horie, MD, PhD, Satoshi Ogiso, MD, PhD, Tomoaki Yoh, MD, PhD, Takahiro Nishio, MD, PhD, Ken Fukumitsu, MD, PhD, Yoichiro Uchida, MD, PhD, Takamichi Ishii, MD, PhD, Etsuro Hatano, MD, PhD

**Table of contents**

Supplemental Table1 and Figure 1 and 2.

**Supplemental TABLE 1**. Characteristics after propensity score matching on preoperative and intraoperative variables (PHLF ≥ B vs < B)

|  | **PHLF < grade B** | **PHLF ≥ grade B** | ***p* value** | **SD** |
| --- | --- | --- | --- | --- |
| **Primary hepatectomy** | **n = 96** | **n = 48** |  |  |
| **Preoperative findings** |  |  |  |  |
| Age > 70 yrs, n (%) | 37 (38.5) | 17 (35.4) | 0.855 | 0.065 |
| Sex, male, n (%) | 82 (85.4) | 40 (83.3) | 0.807 | 0.057 |
| Viral hepatitis, n (%) | 62 (64.6) | 31 (64.6) | 1.000 | <0.001 |
| ALBI score, median (IQR) | -2.57 (-2.99 to -2.38) | -2.60 (-2.82 to -2.36) | 0.498 | 0.092 |
| mALBI grade I , n (%) | 45 (46.9) | 26 (54.2) |  |  |
| grade IIa, n(%) | 31 (32.3) | 13 (27.1) |  |  |
| grade IIb, n(%) | 20 (20.8) | 9 (18.8) |  |  |
| grade III, n(%) | 0 (0.0) | 0 (0.0) | 0.714 |  |
| Serum AFP levels > 200 ng/mL, n (%) | 20 (20.8) | 10 (20.8) | 1.000 | <0.001 |
| Tumor diameter > 5cm, n (%) | 28 (29.2) | 18 (37.5) | 0.346 | 0.177 |
| Multiple tumors, n (%) | 31 (32.3) | 17 (35.4) | 0.712 | 0.066 |
| Poor differentiation, n (%) | 19 (19.8) | 12 (25.0) | 0.522 | 0.125 |
| Cirrhosis (F4), n (%) | 46 (47.9) | 22 (45.8) | 0.861 | 0.042 |
| Macrovascular invasion, n (%) | 14 (14.6) | 5 (10.4) | 0.606 | 0.126 |
| Major hepatectomy, n (%) | 37 (38.5) | 21 (43.8) | 0.591 | 0.106 |
| **Perioperative findings** |  |  |  |  |
| Operation time (min), median (IQR) | 415.0 (319.3 - 490.0) | 419.5 (294.8 - 476.0) | 0.572 | 0.058 |
| Blood loss > 1000ml, n (%) | 40 (41.7) | 19 (39.6) | 0.859 | 0.042 |
| R1 margin, n (%) | 5 (5.3) | 2 (4.3) | 1.000 |  |
| **Recurrence** | **n = 67** | **n = 31** | ***p* value** |  |
| **Findings at recurrence** |  |  |  |  |
| Age > 70 yrs, n(%) | 37 (38.5) | 17 (35.4) | 0.855 |  |
| Serum AFP levels > 200 ng/mL, n (%) | 7 (10.4) | 6 (19.4) | 0.336 |  |
| ALBI score, median (IQR) | -2.67 (-2.82 to -2.42) | -1.94 (-2.17 to -1.73) | <0.001* |  |
| mALBI grade I, n (%) | 37 (55.2) | 4 (12.9) |  |  |
| grade IIa, n (%) | 19 (28.4) | 2 (6.5) |  |  |
| grade IIb, n (%) | 9 (13.4) | 25 (80.6) |  |  |
| grade III, n (%) | 2 (3.0) | 0 (0.0) | < 0.001*† |  |
| Beyond Milan criteria, n (%) | 20 (29.9) | 15 (48.4) | 0.112 |  |
| **Treatment for recurrence** |  |  |  |  |
| Ablation thearpy, n (%) | 26 (38.8) | 8 (25.8) | 0.257 |  |
| Re-resection, n (%) | 17 (25.4) | 2 (6.5) | 0.030* |  |
| Re-resection of IHR, n (%) | 13 (19.4) | 1 (3.2) | 0.034* |  |
| Re-resection of EHR, n (%) | 5 (7.5) | 2 (6.5) | 1.000 |  |
| Transarterial therapy, n (%) | 32 (47.8) | 22 (71.0) | 0.049* |  |
| MTA, n (%) | 23 (34.3) | 10 (32.3) | 1.000 |  |

PHLF, post-hepatectomy liver failure; SD, Standardized Difference; ALBI score, albumin-bilirubin score; mALBI grade, modified albumin-bilirubin grade; IQR, interquartile range; AFP, alpha-fetoprotein; IHR, intrahepatic recurrence; EHR, extrahepatic recurrence; MTA, molecular targeted agent.

*Statistically significant (*p* < 0.05)

† Fisher's exact test; Bonferroni-corrected pairwise comparisons showed significant differences between Grade IIb and Grade I or II

SD: standardized difference


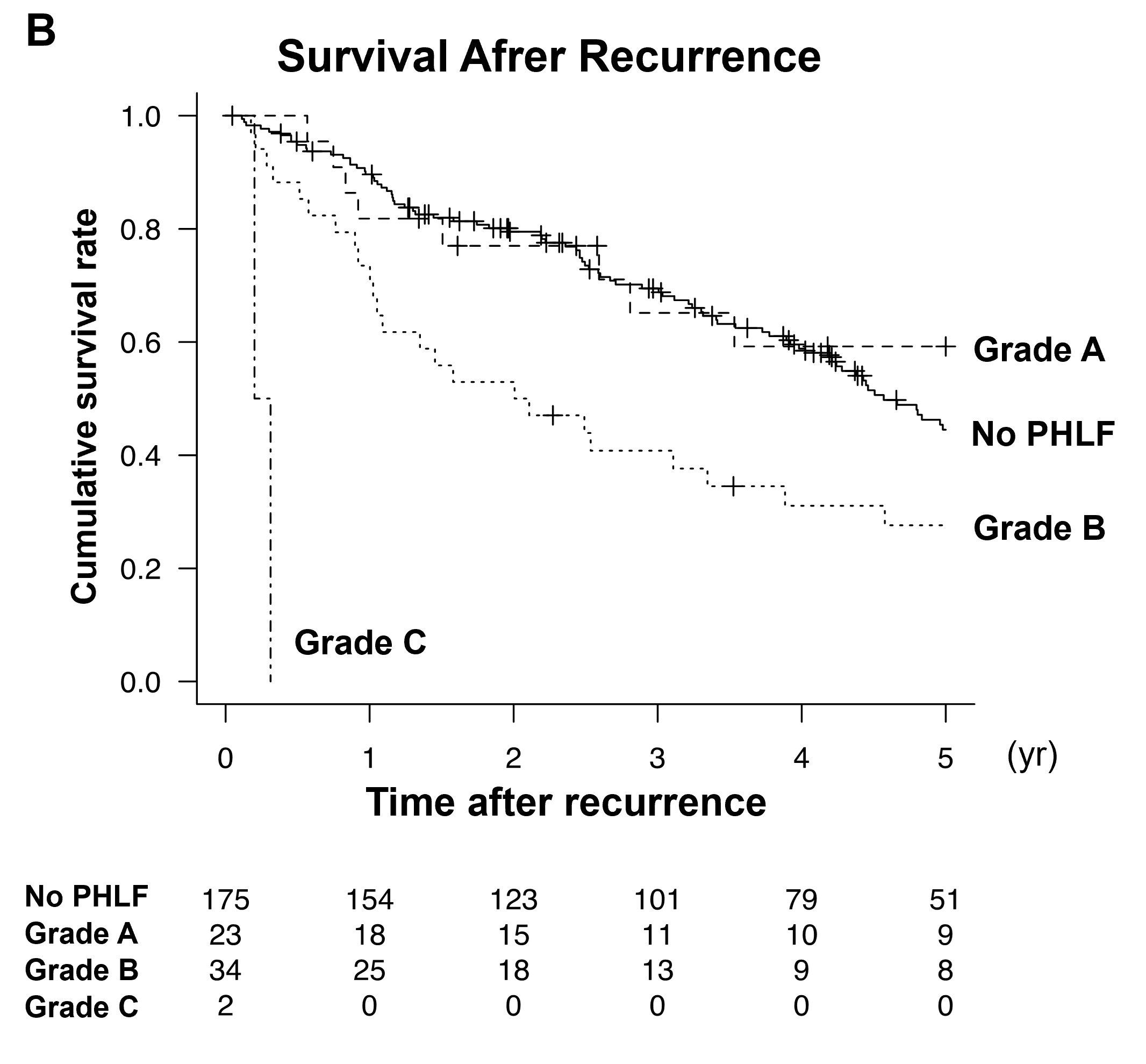

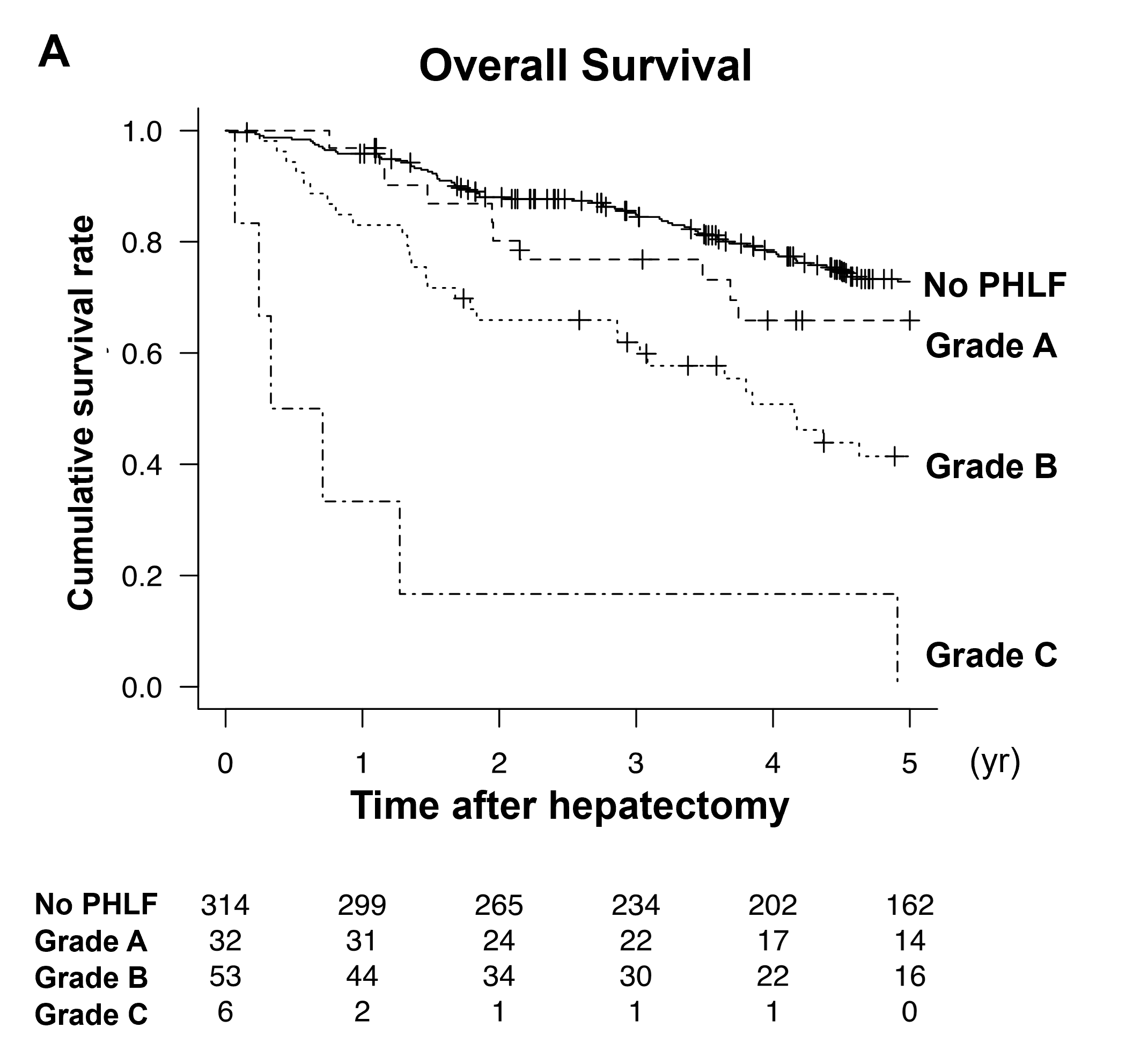


**Supplemental Figure S1.** Overall survival (A) and survival after recurrence (B) according to PHLF grade 0, A, B or C


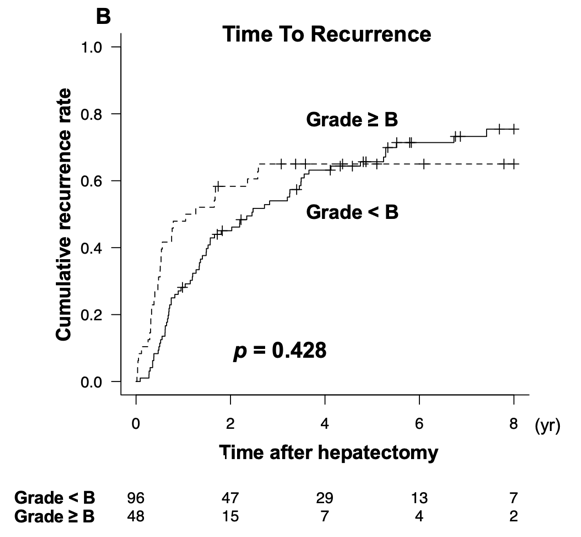

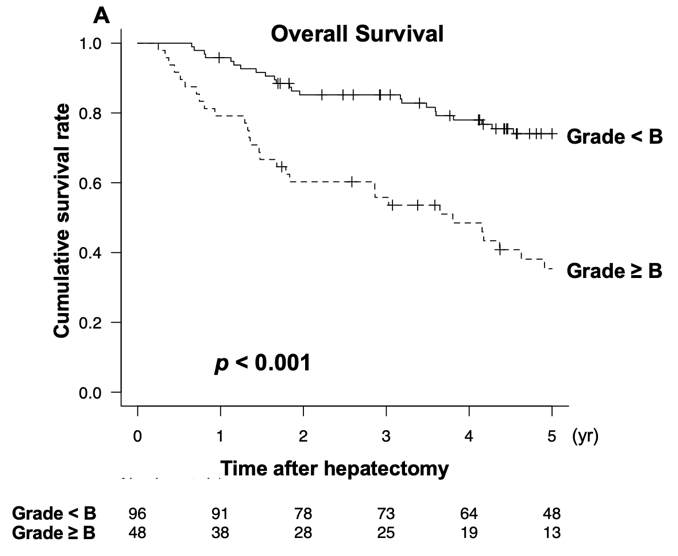


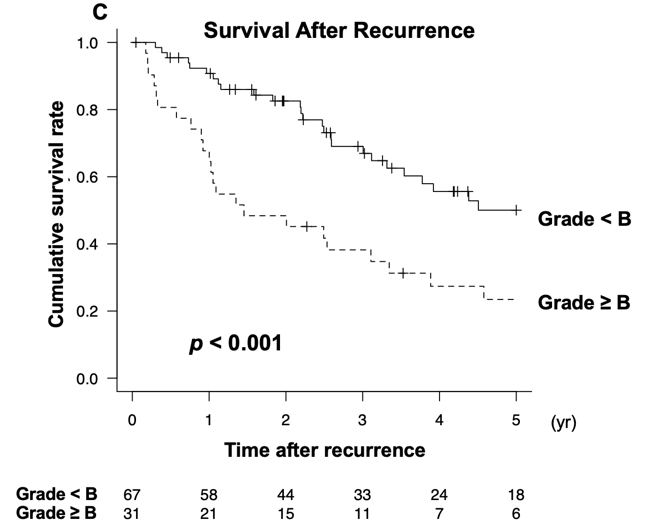


**Supplemental Figure S2**. Overall survival (A), time to recurrence (B), and survival after recurrence (C) after propensity score matching for preoperative and intraoperative variables according to PHLF grade ≥ B or < B
